# *In vivo* evidence for homeostatic regulation of ribosomal protein levels in *Drosophila*

**DOI:** 10.1247/csf.23088

**Published:** 2024-01-11

**Authors:** Daiki Kitamura, Kiichiro Taniguchi, Mai Nakamura, Tatsushi Igaki

**Affiliations:** 1 Laboratory of Genetics, Graduate School of Biostudies, Kyoto University, Kyoto 606-8501, Japan

**Keywords:** ribosomal protein, proteasomal degradation, *Drosophila*

## Abstract

The ribosome is a molecular machine essential for protein synthesis, which is composed of approximately 80 different ribosomal proteins (Rps). Studies in yeast and cell culture systems have revealed that the intracellular level of Rps is finely regulated by negative feedback mechanisms or ubiquitin-proteasome system, which prevents over- or under-abundance of Rps in the cell. However, *in vivo* evidence for the homeostatic regulation of intracellular Rp levels has been poor. Here, using *Drosophila* genetics, we show that intracellular Rp levels are regulated by proteasomal degradation of excess Rps that are not incorporated into the ribosome. By establishing an EGFP-fused Rp gene system that can monitor endogenously expressed Rp levels, we found that endogenously expressed EGFP-RpS20 or -RpL5 is eliminated from the cell when RpS20 or RpL5 is exogenously expressed. Notably, the level of endogenously expressed Hsp83, a housekeeping gene, was not affected by exogenous expression of Hsp83, suggesting that the strict negative regulation of excess protein is specific for intracellular Rps. Further analyses revealed that the maintenance of cellular Rp levels is not regulated at the transcriptional level but by proteasomal degradation of excess free Rps as a protein quality control mechanism. Our observations provide not only the *in vivo* evidence for the homeostatic regulation of Rp levels but also a novel genetic strategy to study *in vivo* regulation of intracellular Rp levels and its role in tissue homeostasis via cell competition.

## Introduction

The ribosome is a pivotal organelle that catalyzes protein synthesis (Moldave, 1985). The macromolecular protein complex of the ribosome consists of approximately 80 ribosomal proteins (Rps) and 4 ribosomal RNAs in eukaryotes (Wilson and Doudna Cate, 2012). Since protein synthesis is an essential cellular process required for the translation of any coding mRNAs, Rps are produced abundantly in every cell and ribosome synthesis accounts for a large energy consumption in the cell (Warner, 1999; Schwarz *et al.*, 2022). Each Rp is required in equimolar amounts for the biosynthesis of ribosomes and the imbalance of each Rp level can cause severe symptoms in animals such as human ribosomopathy or *Drosophila* *Minute* syndrome (Narla and Ebert, 2010; Kongsuwan *et al.*, 1985; Marygold *et al.*, 2007).

It has long been studied how intracellular Rp levels are maintained in a balanced manner. The best studied mechanism is a negative feedback autoregulation of their own expression by Rps (Nomura *et al.*, 1980; Warner and McIntosh, 2009). In *E. coli*, the *Rp* operons encode repressor proteins that bind their own polycistronic mRNA and suppress their translation (Mikhaylina *et al.*, 2021). Studies in yeast have shown that the negative autoregulation of Rp levels is conserved in eukaryotes, where the operon structure is not present (Eng and Warner, 1991; Gabunilas and Chanfreau, 2016; Roy *et al.*, 2020). Intriguingly, in eukaryotes, negative feedback autoregulation of Rp levels has been shown to be achieved by multiple mechanisms such as mRNA degradation (e.g., RpS28B) (Badis *et al.*, 2004), alternative splicing (e.g., RpS26) (Ivanov *et al.*, 2005), and translation inhibition (e.g., RpS3) (Kim *et al.*, 2010). Studies in *C. elegans* have shown that feedback autoregulation of Rp levels can also occur in animals by using RpL10A splicing reporter minigenes or GFP-tagged RpS18 and RpL29 transgenes (Takei *et al.*, 2016; Noma *et al.*, 2017). However, *in vivo* evidence for the feedback autoregulation of endogenous Rp levels has still been missing.

The cellular Rp levels are also regulated at the post-translational level. For instance, blocking rRNA synthesis by Actinomycin D results in continuous Rp synthesis but the newly synthesized Rp is unstable and prone to be degraded (Warner, 1977). Studies in cell culture systems have shown that newly synthesized Rps are rapidly incorporated into the nucleolus and excess Rps are degraded by the proteasome (Lam *et al.*, 2007). Rps that are produced stoichiometrically excess over other Rps are degraded by the ubiquitin-proteasome system (Sung *et al.*, 2016b). Despite many reports on negative regulatory mechanisms of cellular Rp levels, *in vivo* evidence for homeostatic regulation of Rp levels in animals is very poor and it has been elusive how such mechanisms are conserved throughout evolution.

Here, we developed an *in vivo* monitoring system for analyzing homeostatic regulation of cellular Rp levels in *Drosophila* imaginal epithelium, which visualizes endogenously expressed Ribosomal protein S20 (RpS20) or Ribosomal protein L5 (RpL5) as EGFP-fused protein. We found that endogenously expressed EGFP-RpS20 or EGFP-RpL5 protein is eliminated by exogenously expressed RpS20-HA or RpL5-HA protein, respectively. Our data revealed that exogenously expressed Rps are preferentially assembled into the ribosomes and excess Rps that are not incorporated in the ribosomes are degraded by the proteasome. Our observations show *in vivo* evidence for homeostatic regulation of cellular Rp levels in animals, which would provide a new strategy to study the *in vivo* role of Rp regulation in tissue and animal homeostasis.

## Materials and Methods

### Fly strains

*Drosophila melanogaster* strains were raised in vials at 25°C on the standard medium containing glucose, cornmeal, dry yeast, wheat germ, and agar. The sex of larvae used in this study was not differentiated. Fly strains used in this study are as follows: *EGFP-RpS20* (KYOTO Drosophila Stock Center, Kyoto, Japan, #109696), *EGFP-RpL5* (KYOTO Drosophila Stock Center, #109768), *EGFP-Hsp83* (KYOTO Drosophila Stock Center, #109761), *UAS-RpS20-HA* (FlyORF, Zurich, Switzerland, #F000754), *UAS-RpL5-HA* (FlyORF, #F001323), *UAS-RpS3* (Akai *et al.*, 2018), *UAS-Hsp83* (Bloomington Drosophila Stock Center (BDSC), Bloomington, IN, USA, #58468), *UAS-LacZ* (BDSC, #8529), *UAS-p35* (Hay *et al.*, 1994), *nub-Gal4* (BDSC, #42699), *Tub-Gal4* (BDSC, #5138), *UAS-Rpt2-RNAi* (BDSC, #34795), and *UAS-Rpn1-RNAi* (BDSC, #34348). Detailed genotypes of flies used in this study are described in Supplementary Table S1.

### Histology

Wandering third instar larvae were dissected in phosphate buffered saline (PBS) and fixed with 4% paraformaldehyde (PFA) for 5 min on ice followed by 20 min at room temperature. Fixed samples were washed with PBT (PBS + 0.1% Triton X-100) and then soaked in PBT with DAPI (Sigma-Aldrich, St. Louis, MO, USA, #D9542) at 4°C for counterstaining of DNA. For immunostaining with anti-ubiquitin antibody, washed samples were blocked with 5% donkey serum for 30 min and incubated at 4°C overnight with primary antibody (anti-multi ubiquitin, MBL, Tokyo, Japan, #D058-3, 1:500). The samples were then washed, blocked, and incubated with Alexa Fluor 647-conjugated secondary antibody (Invitrogen, Waltham, MA, USA, #A32728, 1:200) for 2 hrs at room temperature. After the incubation, samples were washed and soaked in PBT with DAPI at 4°C. SlowFade Gold Antifade Mountant with DAPI (Invitrogen, #S36939) or anti-fade mounting medium (1 × PBS, 50% Glycerol, 0.2% n-Propyl Gallate, referred to https://www.jacksonimmuno.com/technical/products/protocols) were used as mounting reagents for the slide mounting of samples. Confocal images were taken with TCS SP8 (Leica-Microsystems, Wetzlar, Germany) with the conditions as follows; excitation wavelength: 405 nm (DAPI) and 488 nm (EGFP), filter: 410 nm/480 nm (DAPI) and 492 nm/547 nm (EGFP), and lens: HC PL APO CS2 20×/0.75 DRY. The pouch region was manually outlined and EGFP intensity was measured by Fiji (Schindelin *et al.*, 2012).

### Epifluorescence and light microscopy

Whole larval images were taken using a Leica M165 FC fluorescent microscope.

### Western blot analysis

Three third instar larvae washed with 0.7% NaCl solution were homogenized in 100 μL of Lysis Buffer (15 mM HEPES-KOH pH 7.6, 10 mM KCl, 5 mM MgCl_2_, 0.1 mM EDTA pH 8.0, 10% Glycerol) (Lo Piccolo *et al.*, 2015) using BioMasher II (Nippi, Tokyo, Japan). After centrifugation for 30 sec at 11,000 ×g, lysates were added to 100 μL of Sample Buffer Solution (Nacalai Tesque, Kyoto, Japan, #30566-22) and boiled at 95°C for 5 min. 20 μL of the sample and 5 μL of BLUE Star Prestained Protein-Ladder (Nippon Genetics, Tokyo, Japan, #NE-MWP03) were loaded into each well of SDS-PAGE gels (10%) for electrophoresis and transferred onto PVDF membranes (Millipore, Burlington, MA, USA, #IPVH00010). After blocking with Blocking One (Nacalai Tesque, #03953-95) and washing with Tris Buffered Saline with Tween 20 (TBST), the membranes were incubated with primary antibody (mouse anti-GFP, Clontech, Mountain View, CA, USA, #632381, 1:1000) overnight at room temperature. The membranes were washed with TBST, followed by incubation with horseradish peroxidase (HRP) conjugated secondary antibody (anti-mouse, Cell Signaling Technology (CST), Danvers, MA, USA, #7076, 1:1,000) at room temperature for 2 hrs. The bands were visualized with Chemi-Lumi One Super (Nacalai Tesque, #02230) and detected using ImageQuant LAS4000 (GE Healthcare, Chicago, IL, USA). Then, the membranes were treated with WB Stripping Solution (Nacalai Tesque, #05364-55), and the procedure was repeated for loading control with primary antibody (mouse anti-Tubulin, Sigma-Aldrich, #T5168, 1:5000) and HRP conjugated secondary antibody (anti-mouse). The band intensities were measured by Fiji.

### RNA isolation from larvae and RT-qPCR

The total RNA of each genotype fly was extracted from three larvae and eluted in 13 μL water using NucleoSpin RNA XS (Machery-Nagel, Düren, Germany, #740902.50) according to the manufacturer’s instructions. 11 μL of the eluate was then used to make cDNA with SuperScript IV Reverse Transcriptase (Invitrogen, #18090050) and RT-qPCR was performed using THUNDERBIRD SYBR qPCR Mix (TOYOBO, Osaka, Japan, #QPS-201) on StepOnePlus system (Thermo Fisher Scientific, Waltham, MA, USA). *α-Tubulin at 84B* was used for internal control.

### Polysome Profiling

30 third instar larvae washed with 20% sucrose in PBS were homogenized in 500 μL polysome extraction buffer (20 mM Tris-HCl, pH 7.4, 140 mM KCl, 5 mM MgCl_2_, 1% Triton X-100, 1mM DTT, 100 μg/ml cycloheximide, 800 U/ml RNaseOUT (Invitrogen, #100000840), 2 × Protease Inhibitor Cocktail (Nacalai Tesque, #25955-11)) using BioMasher II (Nippi). The lysates were centrifuged at 16,000 rpm for 15 min at 4°C and the supernatant was transferred to another tube carefully using a syringe to avoid the floating fat content. Finally, extraction was loaded onto 10–50% w/w sucrose gradient (20 mM Tris-HCl, pH 7.4, 140 mM KCl, 5 mM MgCl_2_, 100 μg/ml cycloheximide) and ultracentrifuged at 37,000 rpm for 2.5 hrs in a SW41Ti rotor at 4°C (Optima XE-100 Ultracentrifuge, Beckman, Brea, CA, USA). Gradients were monitored at 254 nm and collected using GradientStation (Biocomp Instruments, Fredericton, NB, Canada) and micro collector AC-5700 (ATTO, Tokyo, Japan). Samples were separated by SDS-PAGE gels (14%) and transferred onto PVDF membrane. The rest of the procedure for Western blot was the same as above. The membranes were stained with primary antibodies (mouse anti-GFP, Clontech #632381, 1:1000 or rat anti-HA, Roche, Basel, Switzerland, #ROAHAHA, 1:1000), and HRP conjugated secondary antibodies (anti-mouse, CST, #7076, 1:1000 or anti-rat, CST, #7077, 1:1000), and detected using Amersham ImageQuant 800 (Cytiva, Tokyo, Japan).

### Statistical analysis

All statistical analyses were performed with EZR (Saitama Medical Center, Jichi Medical University, Saitama, Japan) (Kanda, 2013).

### Primers

Following primers were used for RT-qPCR analyses: endogenously expressed *EGFP-RpS20*, Forward: ATTACCTCCATCAACATCGAGCCCG and Reverse: AGCACGCCAAACTTTTCGAGGTG; *α-Tubulin at 84B*, Forward: TGTCGCGTGTGAAACACTTC and Reverse: AGCAGGCGTTTCCAATCTG (Ponton *et al.*, 2011).

## Results

### Exogenous expression of a ribosomal protein decreases its endogenous protein level

**Fig. 1:**
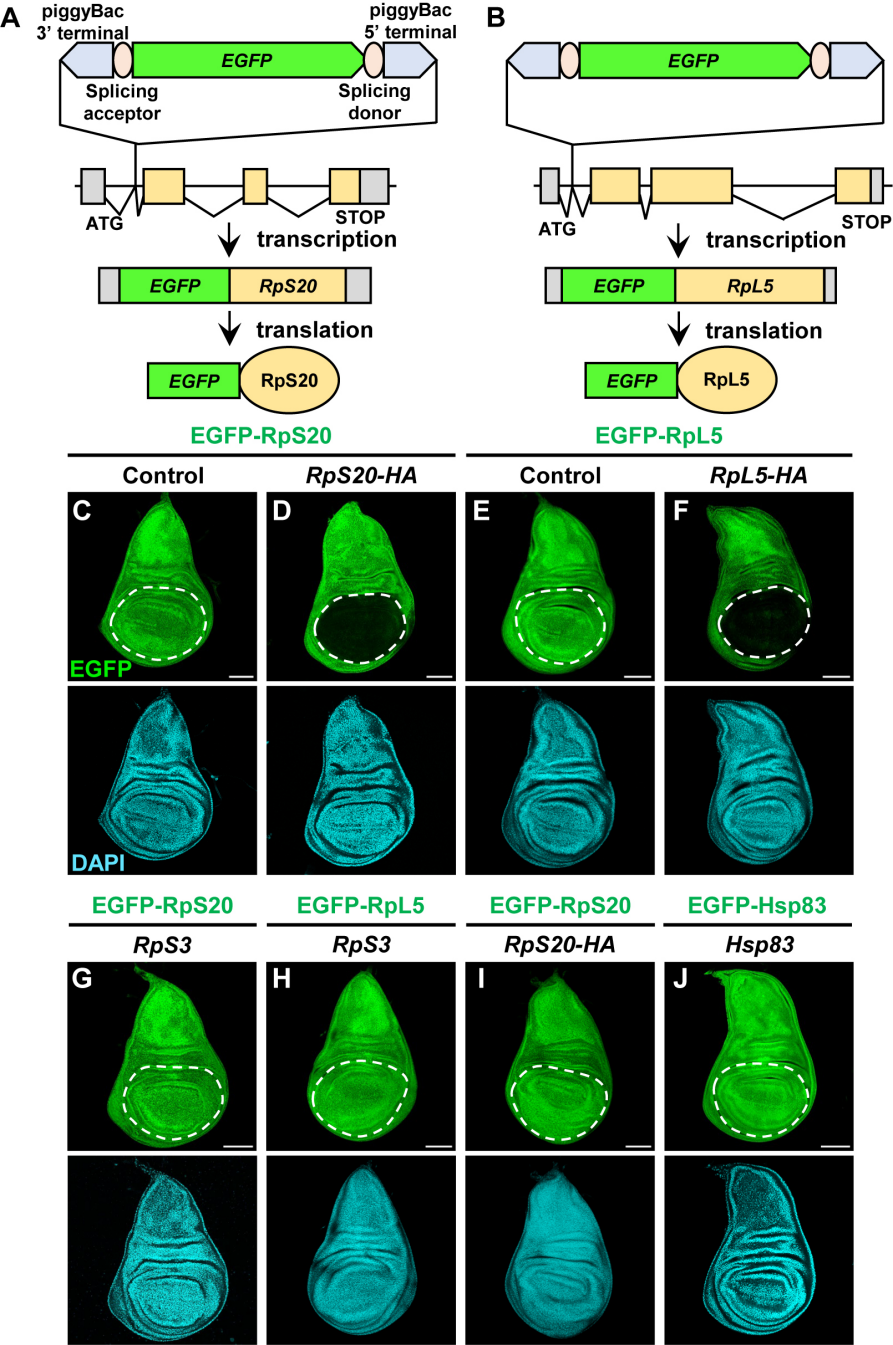
Exogenous expression of a ribosomal protein decreases its endogenous expression (A and B) Schematic drawing of the *EGFP-RpS20* gene cassette and *EGFP-RpL5* gene cassette. Gray box, UTR and yellow box, CDS. (C and D) Wing discs harboring endogenously expressed EGFP-RpS20. *RpS20-HA* is exogenously expressed in the wing pouch region using *nub-Gal4* (D). (E and F) Wing discs harboring endogenously expressed EGFP-RpL5. *RpL5-HA* is exogenously expressed in the wing pouch region (F). (G) Wing disc harboring endogenously expressed EGFP-RpS20. *RpS3* is exogenously expressed in the wing pouch region. (H) Wing disc harboring endogenously expressed EGFP-RpL5. *RpS3* is exogenously expressed in the wing pouch region. (I) Wing disc harboring endogenously expressed EGFP-RpL5. *RpS20-HA* is exogenously expressed in the wing pouch region. (J) Wing disc harboring endogenously expressed EGFP-Hsp83. *Hsp83* is exogenously expressed in the wing pouch region. The regions where the *nub* promoter is activated are indicated by dashed lines. The nucleus is stained with DAPI. Scale bar, 100 μm.

*Drosophila* provides a useful experimental system that visualizes and manipulates gene expressions in living animals. To visualize endogenously expressed Rps in the cell, we used a transgenic fly library of EGFP protein trap lines that have *piggyTrap* transposons containing *EGFP* cDNA flanked by splicing acceptor and donor. In *PBac{EGFP-IV}RpS20^KM0175^* (KYOTO Drosophila Stock Center, #109696) and *PBac{EGFP-IV}RpL5^KM0163^* (KYOTO Drosophila Stock Center, #109768) fly lines, endogenous *RpS20* or *RpL5* gene is fused with EGFP cDNA respectively, resulting in expression of EGFP-fused Rps by the endogenous *Rp* gene promoter (Fig. 1A and 1B). Using these strains, we examined the effect of exogenously expressed Rps on their endogenously expressed EGFP-fused protein level. HA-tagged RpS20 or RpL5 was forced expressed using the Gal4/UAS system by *nubbin-Gal4* (*nub-Gal4*) driver, which induces Gal4 expression specifically in the pouch region of the wing imaginal discs in the larvae. Drastically, forced expression of RpS20-HA or RpL5-HA in the wing pouch abolished the endogenously expressed EGFP-RpS20 or EGFP-RpL5, respectively (Fig. 1D and 1F, compare to Fig. 1C and 1E). Importantly, forced expression of a different Rp, RpS3, did not affect the levels of EGFP-RpS20 (Fig. 1G) or EGFP-RpL5 (Fig. 1H) expression. Similarly, forced expression of RpS20-HA did not affect the expression of EGFP-RpL5 (Fig. 1I). In addition, forced expression of a housekeeping gene *Heat shock protein 83* (*Hsp83*) did not affect the level of EGFP-Hsp83 expression (Fig. 1J). These data suggest that the levels of Rp gene products are specifically regulated in *Drosophila* cells in a homeostatic manner.

### Homeostatic reduction of ribosomal protein levels occurs at the post-transcriptional level

**Fig. 2:**
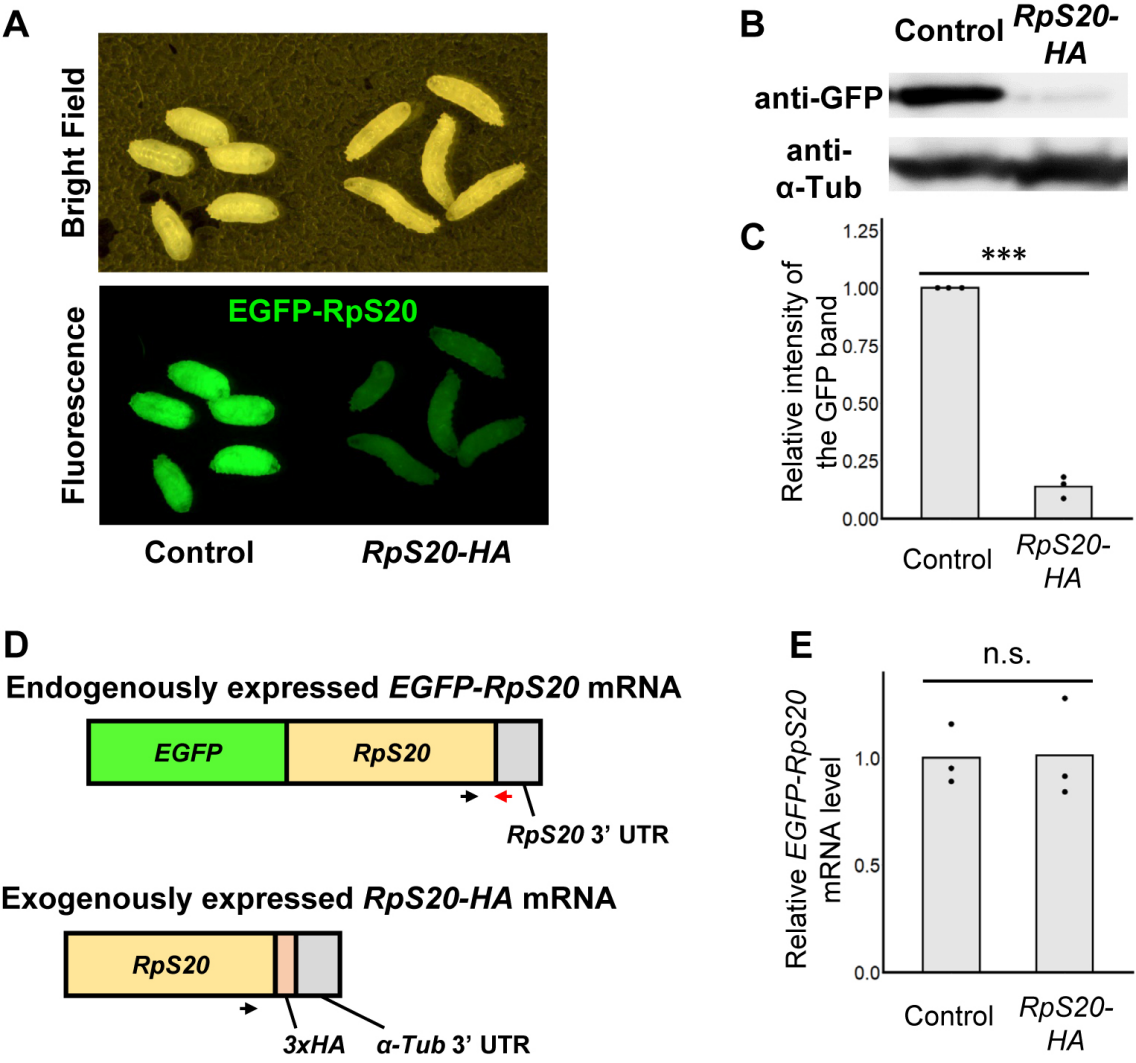
Homeostatic reduction of ribosomal protein levels occurs at the post-transcriptional level (A) Third instar larvae harboring endogenously expressed EGFP-RpS20. *RpS20-HA* is exogenously expressed in the whole body using *Tub-Gal4* (right). (B and C) Larval extracts of each genotype were subjected to Western blot analysis using anti-GFP and anti-α-Tub antibodies. The relative intensity of the GFP bands compared with control was calculated. Black dots show individual measurements and gray bars show the average. n = 3; ***p<0.001; Welch’s t-test. (D) PCR strategies for detecting endogenously expressed *EGFP-RpS20* mRNA. Arrows indicate primers used: Forward (black): ATTACCTCCATCAACATCGAGCCCG and Reverse (red): AGCACGCCAAACTTTTCGAGGTG. (E) Total mRNAs extracted from larvae of each genotype were subjected to RT-qPCR. The relative mRNA levels of *EGFP-RpS20* compared with control were calculated. Black dots show individual measurements and gray bars show the average. n = 3; Welch’s t-test.

We next examined whether the downregulation of endogenous Rp levels by its exogenous expression is a general phenomenon observed in different tissues. Notably, ubiquitous forced expression of RpS20-HA in the whole larva using the *Tubulin-Gal4* (*Tub-Gal4*) driver abolished endogenously expressed EGFP-RpS20 in the whole body (Fig. 2A). The elimination of EGFP-RpS20 protein was further confirmed by Western blot analysis of the whole-larval lysates (Fig. 2B, quantified in 2C). This result also indicated that the elimination of EGFP fluorescence reflects the elimination of the endogenously expressed EGFP-RpS20 protein (Fig. S1). These data suggest that the negative regulation of the Rp levels by its forced expression is a general phenomenon observed in different tissues.

Negative regulation of a gene expression can be achieved at either the transcriptional or post-transcriptional level. We thus examined whether or not the downregulation of endogenous Rp levels by its exogenous expression is caused at the mRNA level. We quantified the endogenously expressed *EGFP-RpS20* mRNA level by RT-qPCR with a primer set that specifically amplifies *EGFP-RpS20* mRNA but not exogenous *RpS20-HA* mRNA (Fig. 2D). We found that forced expression of *RpS20-HA* using *Tub-Gal4* did not affect the level of *EGFP-RpS20* mRNA (Fig. 2E), suggesting that the negative regulation of Rp levels is not caused at the transcriptional level but is mediated by a post-transcriptional mechanism.

### Exogenous expression of Rp downregulates endogenous Rp levels by proteasomal degradation

**Fig. 3:**
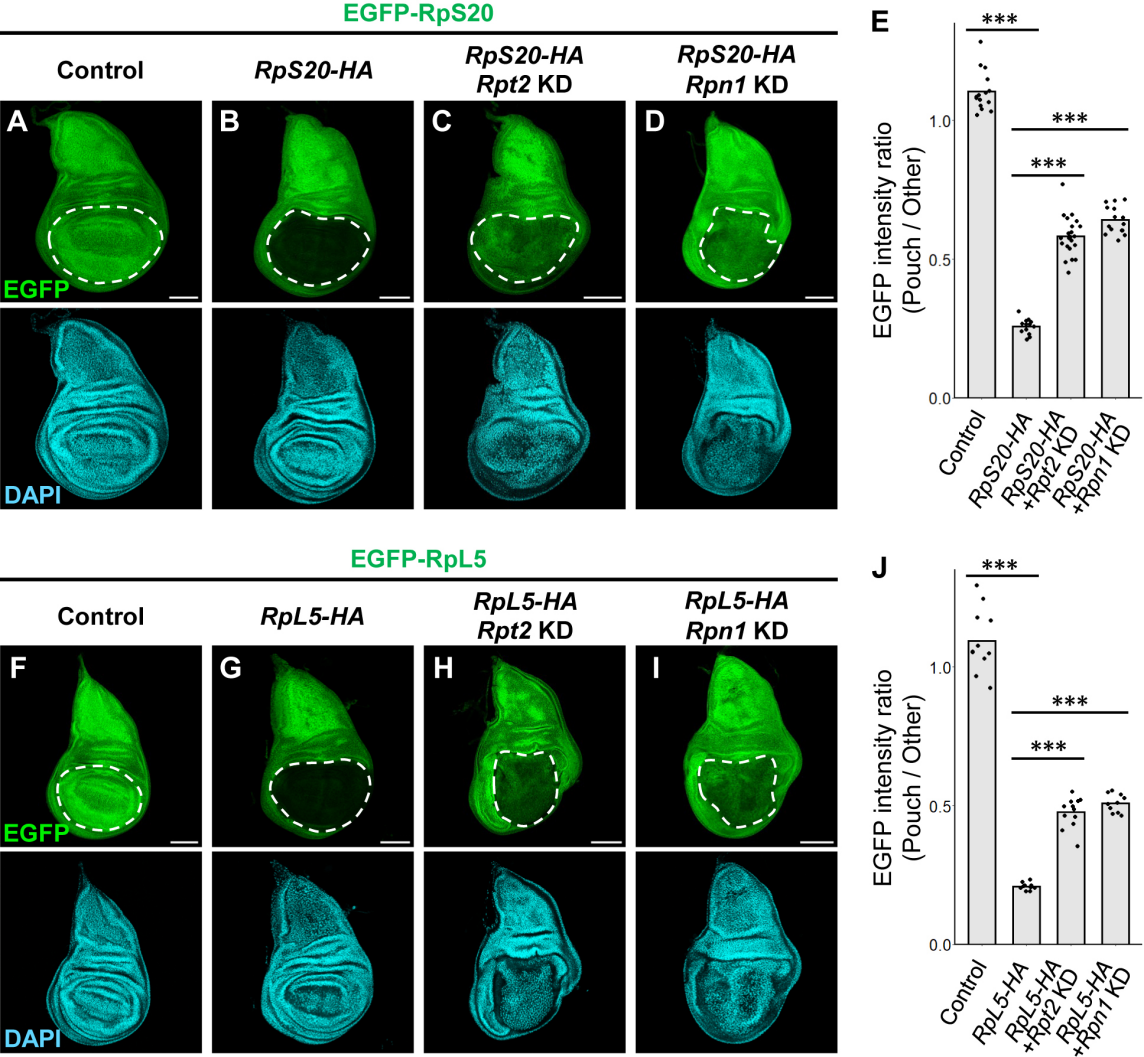
Exogenous expression of Rp downregulates endogenous Rp levels by proteasomal degradation (A–D) Wing discs harboring endogenously expressed EGFP-RpS20. In the wing pouch region, *RpS20-HA* is exogenously expressed (B), and *Rpt2* (C) or *Rpn1* (D) is simultaneously knocked down. (E) Quantification of the ratio of EGFP intensity in the wing pouch region to other regions. Black dots show individual measurements and gray bars show the average. ***p<0.001; Steel-Dwass test. (F–I) Wing discs harboring endogenously expressed EGFP-RpL5. In the wing pouch region, *RpL5-HA* is exogenously expressed (G), and *Rpt2* (H) or *Rpn1* (I) is simultaneously knocked down. (J) Quantification of the ratio of EGFP intensity in the wing pouch region to other regions. Black dots show individual measurements and gray bars show the average. ***p<0.001; Steel-Dwass test.

Post-transcriptional downregulation of a protein can be achieved by either the inhibition of translation or the promotion of protein degradation. We thus tested whether proteasomal degradation contributes to the downregulation of EGFP-RpS20 or EGFP-RpL5 expression when RpS20-HA or RpL5-HA is forced to be expressed. Significantly, blocking proteasome activity by knocking down the proteasomal components *Regulatory particle triple-A ATPase 2* (*Rpt2*) or *Regulatory particle non-ATPase 1* (*Rpn1*) suppressed the downregulation of endogenous EGFP-RpS20 (Fig. 3A–D, quantified in Fig. 3E) and EGFP-RpL5 (Fig. 3F–I, quantified in Fig. 3J) levels when RpS20-HA and RpL5-HA was forced to be expressed, respectively. Inhibition of proteasomal activity by the knockdown of the proteasomal component was confirmed by anti-ubiquitin staining (Fig. S2). These results suggest that exogenous expression of Rp causes downregulation of endogenous Rp levels by proteasomal degradation (Fig. 3E and 3J).

### Endogenous Rps in the ribosomes are replaced by exogenously expressed Rps

**Fig. 4:**
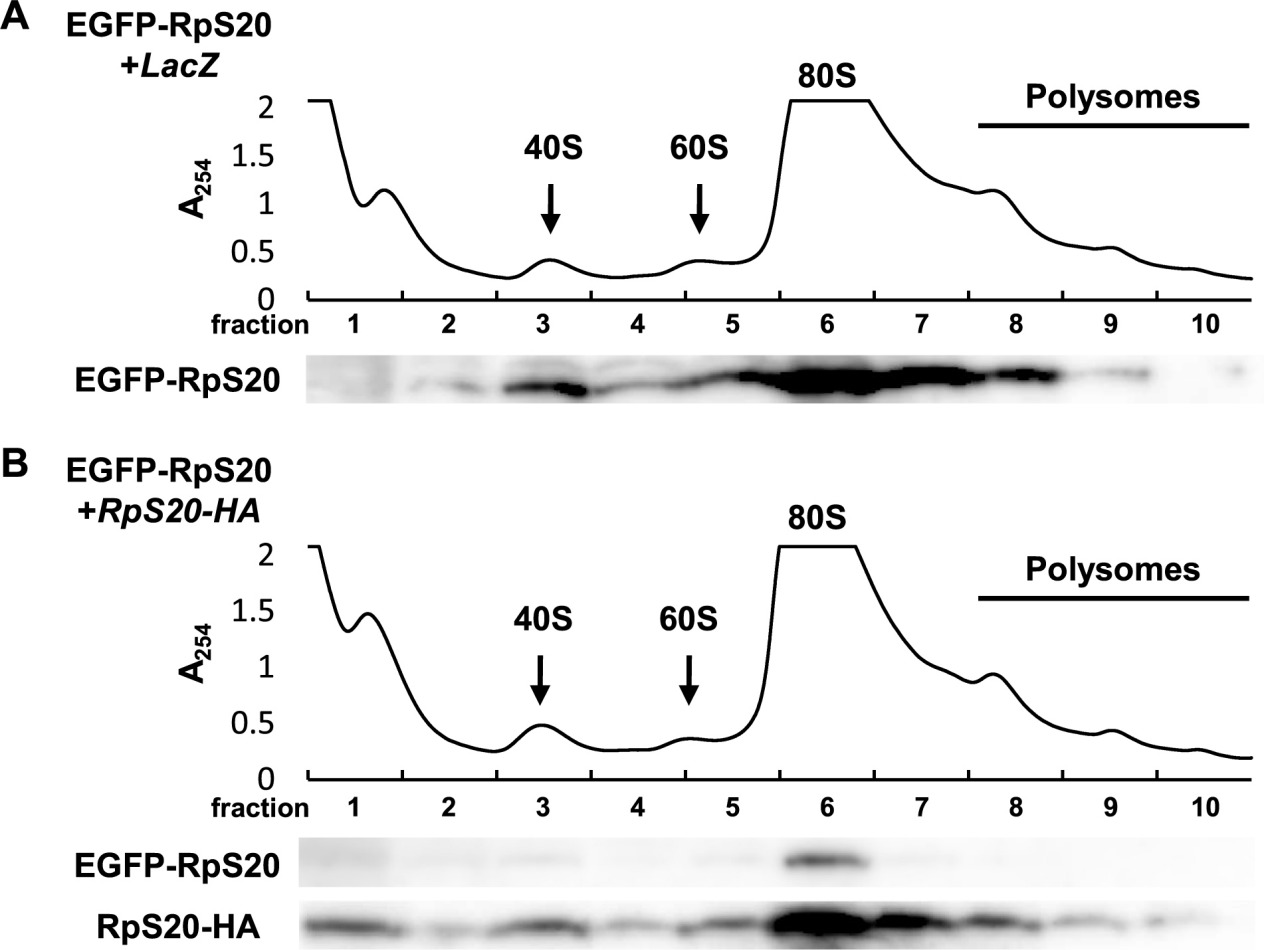
Endogenous Rps in the ribosomes are replaced by exogenously expressed Rps (A and B) Representative polysome profiles of extracts from third instar larvae. Absorbance at 254 nm is shown and the distributions of 40S small subunits, 60S large subunits, 80S monosomes, and polysomes are indicated. Fractionated extracts were subjected to Western blot analysis using anti-GFP and anti-HA antibodies.

From the data presented so far, we hypothesized that exogenously expressed Rp-HA is preferentially incorporated into ribosomes, which causes the endogenously expressed EGFP-Rp to become free ribosomal proteins that are prone to be subjected to proteasomal degradation. To test this hypothesis, we performed polysome profiling and examined the state of endogenous EGFP-RpS20 in the condition where exogenous RpS20-HA was forced to be expressed. In normal conditions, most EGFP-RpS20 proteins were present in 80S ribosomes (Fig. 4A, fraction 6), with a small portion in 40S small subunits (Fig. 4A, fraction 3) and almost no free ribosomal proteins (Fig. 4A, fraction 1) (Fig. 4A). Notably, forced expression of RpS20-HA significantly reduced the amount of EGFP-RpS20 in the 40S small subunits (Fig. 4B, fraction 3) and 80S ribosomes (Fig. 4B, fraction 6). This was further confirmed by the increased amount of RpS20-HA incorporated in the 40S small subunits and 80S ribosomes (Fig. 4B). The amount of free RpS20 was increased in the cell when RpS20-HA was forced to be expressed (Fig. 4B, fraction 1), suggesting that the proteasome is overloaded with the overproduced Rps.

## Discussion

**Fig. 5:**
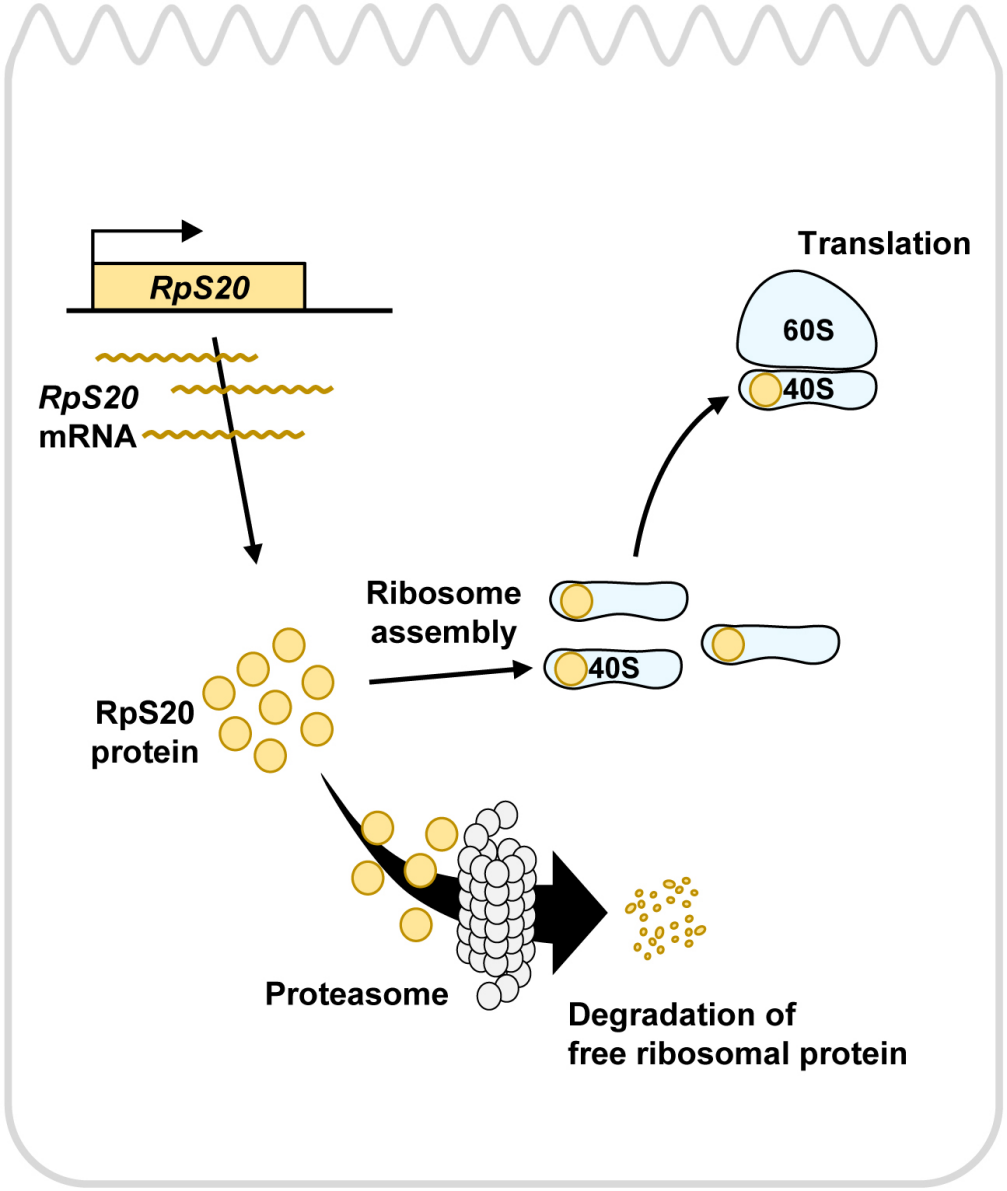
A model for homeostatic regulation of intracellular ribosomal protein levels by proteasomal degradation in *Drosophila* Equimolar amounts of RpS20 proteins with other Rps are used for ribosome assembly and free RpS20 that are not incorporated into ribosomes are rapidly degraded by proteasomal degradation.

In this study, we have established an experimental system that visualizes a negative regulation of the amount of endogenously expressed RpS20 or RpL5 proteins in *Drosophila* epithelia. This *in vivo* monitoring system allows us to investigate the homeostatic regulation of intracellular Rps in normal cells of living organisms, but not in immortalized cultured cells, as well as to perform genetic studies to understand the role of Rp level regulation in tissue homeostasis via Rp-related biological phenomena such as cell competition (see below). Genetic analysis using this system revealed that exogenously expressed RpS20 or RpL5 reduces endogenously expressed RpS20 or RpL5 proteins in the cell. Mechanistically, forced expression of Rps in the cell causes excess free Rps that are not incorporated into the ribosomes, which are prone to be subjected to proteasomal degradation (Fig. 5). Importantly, forced expression of a housekeeping gene product Hsp83 did not cause such a homeostatic negative regulation, suggesting that the amount of Rps in the cells is finely regulated by a specific mechanism.

A phenomenon that overproduced Rps are rapidly degraded in the cell has previously been reported in *S. cerevisiae* (Gorenstein and Warner, 1977), but the underlying mechanism remained unknown until recently. Eukaryotic cells possess a protein quality control (PQC) mechanism that degrades cytosolic unfolded proteins and thus prevents proteotoxic stress. It has recently been shown that “orphan” subunits including Rps that failed to be incorporated into the protein complex are subjected to PQC (Juszkiewicz and Hegde, 2018; Kong *et al.*, 2021). When supernumerary Rps are present in the cell for some reasons such as rRNA deficiency or extra copies of Rp genes, a conserved HECT E3 enzyme Tom1 (yeast) or HUWE1 (mammals) ubiquitinates excess Rps, leading to proteasomal degradation, a phenomenon called ERISQ (excess ribosomal protein regulation) (Sung *et al.*, 2016a, 2016b). It has also been shown by *in vitro* assay that UBE2O, a ubiquitin-conjugating enzyme involved in cytosolic degradation of orphan proteins that are not assembled into protein complexes, targets Rps (Yanagitani *et al.*, 2017). UBE2O recognizes juxtaposed basic and hydrophobic patches, which are often seen in Rps, and degrades Rps in the cytoplasm. Newly synthesized Rps form insoluble aggregates in yeast cells that are treated with a proteasome inhibitor, implying that the degradation of excess Rps by PQC is important for preventing proteotoxic stress (Sung *et al.*, 2016a, 2016b). Our data provide the *in vivo* evidence that the proteasomal degradation plays a crucial role in maintaining Rps homeostasis in flies.

The fact that the intracellular Rp levels are strictly regulated by multiple mechanisms suggests that maintaining all Rps in equimolar amounts is crucial for the cell. Indeed, a series of *Drosophila* mutants heterozygous for a ribosomal protein gene called ‘*Minute*’ mutants commonly exhibit thin-bristles and slow-growth phenotypes (Marygold *et al.*, 2007). Notably, while *Minute* wing imaginal discs or eye imaginal discs develop into essentially normal wings or eyes respectively, *Minute* cells are actively eliminated from the imaginal epithelium when surrounded by wild-type cells, a phenomenon called cell competition (Morata and Ripoll, 1975; Nagata and Igaki, 2018). The physiological role of cell competition is still largely elusive, but it is possible that cell competition optimizes tissue fitness by eliminating abnormal cells that have an imbalance in intracellular Rp levels. This imbalance activates the PQC machinery and may cause proteotoxic stress if PQC is overwhelmed. Notably, proteotoxic stress has been shown to be a driver of cell competition (Baumgartner *et al.*, 2021). Further studies on the mechanisms of homeostatic regulation of Rp levels and its role in cell competition would clarify how intracellular regulations of Rp levels contribute to tissue and animal homeostasis.

## Funding

This work was financially supported in part by grants from the MEXT/JSPS KAKENHI (21H05284 and 21H05039) to T.I, AMED-CREST, Japan Agency for Medical Research and Development (22gm1710002h0001) to T.I, the Takeda Science Foundation to T.I, and the Naito Foundation to T.I.
