# Supplementary material for: In vivo evidence for homeostatic regulation of ribosomal protein levels in Drosophila
Source: Cell Struct Funct. 2024 Jan 11;49(1):11–20. doi: 10.1247/csf.23088 (PMC11496781; doi:10.1247/csf.23088)
Supplement: Supplementary file 1 — Supplementary Materials [file csf_49_23088_1.zip › 49_23088_1.pdf]

Fig. S1

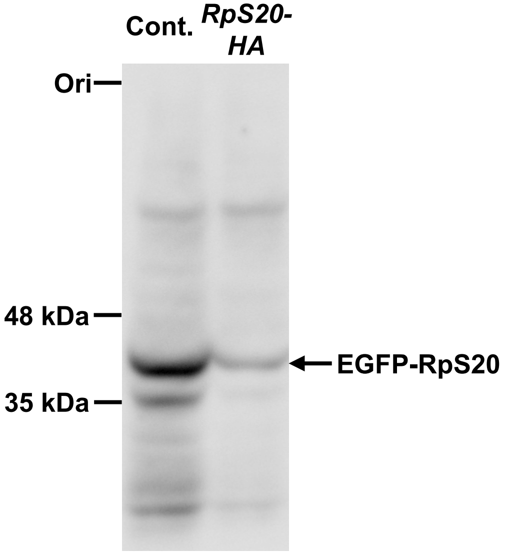

Endogenously-expressed EGFP-RpS20 is eliminated by exogenous expression of *RpS20-HA*

Larval extracts of each genotype were subjected to Western blot analysis using anti-GFP antibody. Bands are detected at the predicted molecular weight of EGFP-RpS20. The positions of protein molecular weight markers are indicated on the left.
