# Supplementary material for: In vivo evidence for homeostatic regulation of ribosomal protein levels in Drosophila
Source: Cell Struct Funct. 2024 Jan 11;49(1):11–20. doi: 10.1247/csf.23088 (PMC11496781; doi:10.1247/csf.23088)
Supplement: Supplementary file 1 — Supplementary Materials [file csf_49_23088_1.zip › 49_23088_2.pdf]

Fig. S2

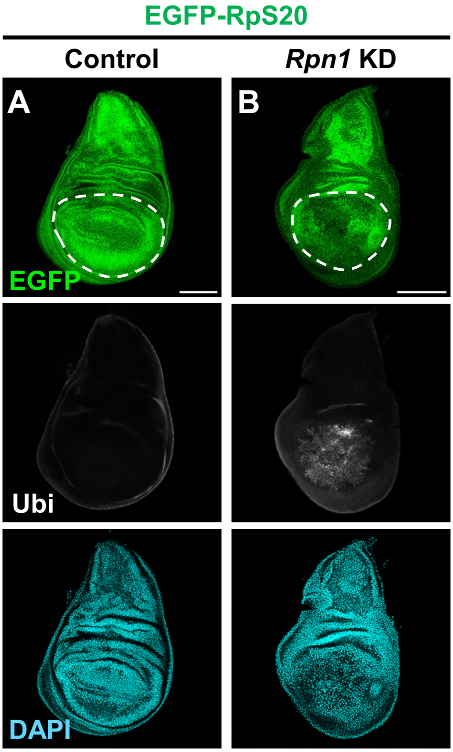

Knockdown of the proteasomal component inhibits proteasomal activity
(A and B) Wing discs harboring endogenously expressed EGFP-RpS20 were stained with anti-ubiquitin antibody. *Rpn1* is knocked down in the wing pouch region (dashed lines) (B). The nucleus is stained with DAPI. Scale bar, 100 μm.
