# Supplementary material for: In vivo evidence for homeostatic regulation of ribosomal protein levels in Drosophila
Source: Cell Struct Funct. 2024 Jan 11;49(1):11–20. doi: 10.1247/csf.23088 (PMC11496781; doi:10.1247/csf.23088)
Supplement: Supplementary file 1 — Supplementary Materials [file csf_49_23088_1.zip › 49_23088_3.docx]

**Supplementary Table S1**

**Genotypes of flies used in the experiments**

Fig.1

(C) +/+; *EGFP-RpS20*/+

(D) *nub-Gal4*/+; *EGFP-RpS20*, *UAS-RpS20-HA*/+

(E) *EGFP-RpL5*/+; +/+

(F) *nub-Gal4*/*EGFP-RpL5*; *UAS-RpL5-HA*/+

(G) *nub-Gal4*/+; *EGFP-RpS20*/*UAS-RpS3*

(H) *nub-Gal4*/*EGFP-RpL5*; *UAS-RpS3*/+

(I) *nub-Gal4*/*EGFP-RpL5*; *UAS-RpS20-HA*/+

(J) *nub-Gal4*/+; *EGFP-Hsp83*/*UAS-Hsp83*

Fig.2

(A, left) +/+; *EGFP-RpS20*, *UAS-RpS20-HA*/*TM6B*

(A, right) +/+; *EGFP-RpS20*, *UAS-RpS20-HA*/*Tub-Gal4*

(B, left) +/+; *EGFP-RpS20*, *UAS-RpS20-HA*/*TM6B*

(B, right) +/+; *EGFP-RpS20*, *UAS-RpS20-HA*/*Tub-Gal4*

(C, left) +/+; *EGFP-RpS20*, *UAS-RpS20-HA*/*TM6B*

(C, right) +/+; *EGFP-RpS20*, *UAS-RpS20-HA*/*Tub-Gal4*

(E, left) +/+; *EGFP-RpS20*, *UAS-RpS20-HA*/*TM6B*

(E, right) +/+; *EGFP-RpS20*, *UAS-RpS20-HA*/*Tub-Gal4*

Fig.3

(A) *UAS-p35*/+; *nub-Gal4*/+; *EGFP-RpS20*/+

(B) *UAS-p35*/+; *nub-Gal4*/+; *EGFP-RpS20*, *UAS-RpS20-HA*/+

(C) *UAS-p35*/+; *nub-Gal4*/+; *EGFP-RpS20*, *UAS-RpS20-HA*/*UAS-Rpt2-RNAi*

(D) *UAS-p35*/+; *nub-Gal4*/+; *EGFP-RpS20*, *UAS-RpS20-HA*/*UAS-Rpn1-RNAi*

(E, lane 1) *UAS-p35*/+; *nub-Gal4*/+; *EGFP-RpS20*/+

(E, lane 2) *UAS-p35*/+; *nub-Gal4*/+; *EGFP-RpS20*, *UAS-RpS20-HA*/+

(E, lane 3) *UAS-p35*/+; *nub-Gal4*/+; *EGFP-RpS20*, *UAS-RpS20-HA*/*UAS-Rpt2-RNAi*

(E, lane 4) *UAS-p35*/+; *nub-Gal4*/+; *EGFP-RpS20*, *UAS-RpS20-HA*/*UAS-Rpn1-RNAi*

(F) *UAS-p35*/+; *nub-Gal4*/*EGFP-RpL5*; +/+

(G) *UAS-p35*/+; *nub-Gal4*/*EGFP-RpL5*; *UAS-RpL5-HA*/+

(H) *UAS-p35*/+; *nub-Gal4*/*EGFP-RpL5*; *UAS-RpL5-HA*/*UAS-Rpt2-RNAi*

(I) *UAS-p35*/+; *nub-Gal4*/*EGFP-RpL5*; *UAS-RpL5-HA*/*UAS-Rpn1-RNAi*

(J, lane 1) *UAS-p35*/+; *nub-Gal4*/*EGFP-RpL5*; +/+

(J, lane 2) *UAS-p35*/+; *nub-Gal4*/*EGFP-RpL5*; *UAS-RpL5-HA*/+

(J, lane 3) *UAS-p35*/+; *nub-Gal4*/*EGFP-RpL5*; *UAS-RpL5-HA*/*UAS-Rpt2-RNAi*

(J, lane 4) *UAS-p35*/+; *nub-Gal4*/*EGFP-RpL5*; *UAS-RpL5-HA*/*UAS-Rpn1-RNAi*

Fig.4

(A) *UAS-LacZ*/+; *EGFP-RpS20*/*Tub-Gal4*

(B) +/+; *EGFP-RpS20*, *UAS-RpS20-HA*/*Tub-Gal4*

Fig.S1

(left) +/+; *EGFP-RpS20*, *UAS-RpS20-HA*/*TM6B*

(right) +/+; *EGFP-RpS20*, *UAS-RpS20-HA*/*Tub-Gal4*

Fig.S2

(A) *nub-Gal4*/+; *EGFP-RpS20*/+

(B) *nub-Gal4*/+; *EGFP-RpS20*/*UAS-Rpn1-RNAi*
